# Long-Term Health Outcomes of Korean Adults With Classic Congenital Adrenal Hyperplasia Due to 21-Hydroxylase Deficiency

**DOI:** 10.3389/fendo.2021.761258

**Published:** 2021-10-12

**Authors:** Seung Gyun Lim, Young Ah Lee, Han Na Jang, Sung Hye Kong, Chang Ho Ahn, Sang Wan Kim, Choong Ho Shin, Jung Hee Kim

**Affiliations:** ^1^ Department of Internal Medicine, Seoul National University Hospital, Seoul, South Korea; ^2^ Department of Internal Medicine, Seoul National University College of Medicine, Seoul, South Korea; ^3^ Department of Pediatrics, Seoul National University Children’s Hospital, Seoul National University College of Medicine, Seoul, South Korea; ^4^ Department of Internal Medicine, Seoul National University Bundang Hospital, Seongnam, South Korea; ^5^ Department of Internal Medicine, Seoul Metropolitan Government Seoul National University Boramae Medical Center, Seoul, South Korea

**Keywords:** adrenal hyperplasia, congenital, dyslipidemia, hyperglycemia, hypertension, obesity

## Abstract

There is a lack of studies regarding the long-term outcomes of Asian adults with classic congenital adrenal hyperplasia (CAH) due to 21-hydroxylase deficiency. We hypothesized that adults with CAH are at higher metabolic risk than their age-, and sex-matched controls. We further investigated the long-term health outcome-related factors in adults with CAH. We compared metabolic risk between adults with CAH (71 men, 93 women) and age-, and sex-matched controls (190 men, 261 women) from the Korean National Health and Nutrition Examination Survey data. The presence of obesity, testicular adrenal rest tumors (TARTs), and menstrual irregularity was assessed. Hormone status and treatment regimens were compared according to the presence of adverse outcomes. The median age was 27.0 y and 28.0 y for men and women, respectively. Adults with CAH had a higher waist circumference (88.0 vs. 82.3 cm in men, and 83.5 *vs*. 72.3 cm in women), and blood pressure (125.0 vs. 113.0 mmHg in men, and 120.0 *vs*. 104.0 mmHg in women) than age- and sex-matched controls (*P*<0.05 for all). The 2.7-fold increased risk for hypertension (men) and 2.0-fold increased risk for obesity (women) was significant in patients with CAH (*P*<0.05 for both). Obese adults with CAH showed significantly higher adrenal limb thicknesses (men) and 17-hydroxyprogesterone and dehydroepiandrosterone sulfate levels (women) (*P*<0.05 for both). TARTs occurred in 58.1% of men and did not differ by hormone or treatment regimen. Irregular menstruation was observed in 57.1% of women, with higher dehydroepiandrosterone sulfate levels in those with irregular periods. Adults with CAH had a higher metabolic risk than the general population. Poor disease control may increase their risk of metabolic morbidity and menstrual irregularity.

## Introduction

Congenital adrenal hyperplasia (CAH) refers to a group of genetic disorders characterized by defective steroidogenesis due to enzyme deficiency. The most common form of CAH, 21-hydroxylase deficiency, affects approximately 1:15,000 live births (1, 2). A 21-hydroxylase deficiency results in glucocorticoid deficiency and an increase in pituitary adrenocorticotropin (ACTH) secretion, leading to adrenal androgen excess and adrenal hyperplasia. Depending on disease severity, CAH due to 21-hydroxylase deficiency is classified into classic (salt-wasting and simple virilizing) and non-classic forms (1). The early diagnosis of 21-hydroxylase deficiency through newborn screening test using 17-hydroxyprogesterone (17-OHP) has decreased mortality and morbidity rates, leading to increased interest in improving long-term health outcomes in adulthood (1, 2).

Several studies have reported adverse outcomes in adults with CAH. In the United Kingdom Congenital Adrenal Hyperplasia Adult Study Executive (CaHASE), 203 adults with CAH were significantly shorter and had a higher body mass index (BMI) compared to the health survey data (3). In a Swedish study, based on disease codes, 360 adults with CAH had an approximately four-fold higher risk of having any cardiovascular and metabolic disorders than matched controls (4). In a cross-sectional study of the National Institutes of Health (NIH) and French cohort, the prevalence of obesity was common, ranging from 35% to 44% (5, 6), although the latter two studies did not compare the patients’ metabolic risk with that of healthy controls. In a European multicenter study, 226 adults with CAH were a higher risk for hypertension, dyslipidemia, and cardiovascular disease but not type 2 diabetes (7). A recent meta-analysis including children and adults with CAH demonstrated that these patients had a high prevalence of cardiovascular and metabolic risk factors (8).

The two main goals for CAH management are to replace the deficient hormones for adrenal insufficiency and control androgen excess. However, it is challenging to balance glucocorticoid doses because supraphysiologic doses of glucocorticoids are usually required to suppress androgen excess. It remains controversial whether over- or undertreatment is more harmful to the metabolic and cardiovascular health of patients with CAH (3, 5, 6, 9–11). Moreover, the relationship between testicular adrenal rest tumors (TARTs) and disease control remains unclear (5, 6, 12).

Most related studies have not performed a sex-stratified analysis and rarely evaluated adrenal morphology such as hyperplasia or thinning (6, 13). In Asia, few studies have examined the determining factors for adverse outcomes in adults with CAH. In this context, we hypothesized that adults with CAH are at a higher risk of metabolic morbidity than their age- and sex-matched controls in the Asian general population. Furthermore, we aimed to investigate sex-specific indicators related to adverse health outcomes by focusing on metabolic morbidity, TARTs, and menstrual irregularity in adults with CAH.

## Materials and Methods

### Study Subjects

Among the 233 adults with CAH aged over 20 years at the last follow-up visit between 2000 and 2020 at Seoul National University Hospital, those with non-classic CAH (n = 19) or for whom laboratory findings or an appropriate medical history was lacking (n = 50) were excluded. Finally, we included 164 adults with classic 21-hydroxylase deficiency. Of the 71 men and 93 women included in this study 42 (59.2%) men and 34 (36.6%) women had salt-wasting form. We diagnosed patients with CAH based on clinical and biochemical data since the genetic testing for CYP21A2 mutation was only done in 34 of 164 patients. All patients were diagnosed clinically since neonatal screening was first introduced in 2006 in Korea. For comparison, age- and sex-matched controls (190 men, 261 women) were included in this study, with a 1:3 case to control ratio from the nationwide representative survey database (Korean National Health and Nutrition Examination Survey [KNHANES] 2015) (14).

### Clinical, Imaging, and Biochemical Data

We retrospectively reviewed the patients’ electronic medical records and retrieved the data from the last follow-up visit. BMI was calculated as body weight divided by height squared (kg/m^2^). Waist circumference was measured at the level of the umbilicus with the subject standing and breathing normally while wearing light clothing. Blood pressure was measured twice on different days using an automated technique while the subjects were in a seated position after a 20-min rest. Body composition data, such as lean mass and fat mass, were obtained from a bioimpedance analyzer (Inbody720^®^; Inbody Co. Korea).

Obesity was defined as a BMI > 25 kg/m^2^ (15). Dyslipidemia was defined as the use of lipid-lowering agents or having an abnormal lipid panel (total cholesterol ≥ 240 mg/dL, LDL cholesterol ≥ 160 mg/dL, triglycerides ≥ 200 mg/dL, or HDL cholesterol < 40 mg/dL). The presence of hyperglycemia included diabetes mellitus and prediabetes. Diabetes mellitus was defined as an HbA1c ≥ 6.5% or the use of any oral anti-diabetic drugs or insulin therapy. Prediabetes was determined as an HbA1c value between 5.7% and 6.4% or a fasting plasma glucose ≥100 mg/dL. Subjects taking any antihypertensive medications or systolic blood pressure ≥ 130 mmHg and/or diastolic blood pressure ≥ 85 mmHg in repeated measurements were considered to have hypertension (16). In men, TARTs were identified using testicular sonography. In women, the regularity of menstruation was classified into two categories: regular (21–35 days) or irregular (oligomenorrhea/amenorrhea).

Computed tomography was performed to evaluate the morphology of the adrenal glands. The examiner was blinded to the patients’ history. The widths of the medial and lateral adrenal limbs were measured as the maximum width of the limbs perpendicular to the long axis (17). The thickness of the adrenal limb was defined as the mean medial and lateral limb widths. The current glucocorticoid and mineralocorticoid (fludrocortisone) regimen was identified for each patient. The daily glucocorticoid dose was calculated based on glucocorticoid type and daily dose. Glucocorticoid doses were calculated based on the anti-inflammatory equivalent dose compared to hydrocortisone (30 mg hydrocortisone = 7.5 mg prednisolone).

Morning fasting blood samples were taken before steroid medications were administered. Laboratory tests included hemoglobin A1c (HbA1c), plasma glucose, total cholesterol, triglycerides, high-density lipoprotein (HDL) cholesterol, low-density lipoprotein (LDL) cholesterol, 17-hydroxyprogesterone (17-OHP), total testosterone, dehydroepiandrosterone sulfate (DHEAS), plasma renin activity, and adrenocorticotropin (ACTH).

### Biochemical assays

Serum 17-OHP and DHEAS levels were measured using a radioimmunoassay (RIA) CT kit (Asbach Medical Products GmbH, Germany), with intra- and inter-assay CVs of 4.6–6.8% and 7.7–8.8% for 17-OHP (reference range, 0.11-5.00 ng/mL) and 3.6–5.9% and 6.5% for DHEAS (reference range, 1187-4289 ng/mL), respectively. Serum total testosterone was measured using a TESTO-CT2 kit (Cisbio Bioassays, Saclay, France) with intra- and inter-assay CVs of 3.1–8.9% and 5.2–11.6%, respectively (reference range, 2.7-10.7 ng/mL in men, and 0-1.0 ng/mL in women). Plasma renin activity was measured using a PRA RIA kit (TFB, Inc.), with intra- and inter-assay CVs of 3.8% and 6.7%, respectively (reference range, 0.32-1.84 ng/mL/hr). Plasma ACTH was measured using an immunoradiometric assay (Cisbio Bioassays) with a reference range of 10.0–60.0 pg/mL. The intra- and inter-assay CVs of the ACTH used were 3.7% and 3.8%, respectively.

### Statistical Analysis

Data are shown as number (percentage) for categorical variables and mean ± standard deviation or median (interquartile range) for continuous variables based on the results of the normality test. The normality test was performed using the Shapiro-Wilk test. Categorical variables were analyzed using the chi-squared test. Continuous variables were compared using Student’s t-test or the Mann-Whitney U test according to the distribution of normality. Logistic regression models were constructed to assess the metabolic risk of patients with CAH by sex. To adjust for the effect of age and BMI, we performed a multivariate logistic regression analysis, and age and BMI-adjusted odds ratio (OR) and 95% confidence interval (95% CI) are presented. Statistical significance was set at P < 0.05. All statistical analyses were performed using R Statistical Software (version 4.0.3).

## Results

### Comparison of Metabolic Risk Between Patients with CAH and Healthy Controls

**Table 1** compares the clinical and biochemical characteristics of patients with CAH and age- and sex-matched controls by sex. The median age for men and women was 27.0 and 28.0 years, respectively. The men with CAH (n = 71) exhibited a shorter stature (P < 0.001), higher weight, and greater waist circumference (P = 0.005 for both) than the control men (n = 190) without a BMI difference. Fasting plasma glucose and blood pressure were higher in the men with CAH (P < 0.05), but HbA1c was similar between the two groups. Although total cholesterol, triglyceride, and LDL cholesterol levels were similar between the two groups, HDL cholesterol was higher in the men with CAH than in the control men (P < 0.001).

**Table 1:** Comparison of clinical characteristics between CAH patients and age- and sex-matched control.

Variables	CAH men	Control men	*P*	CAH women	Control women	P
	(N = 71)	(N = 190)		(N = 93)	(N = 261)	
Age, years	27.0 [23.0;33.0]	27.0 [23.0;33.0]	0.918	28.0 [23.0;36.0]	28.0 [23.0;36.0]	0.937
Height, cm	163.8 [157.6;167.9]	174.7 [170.5;177.8]	<0.001	152.8 [147.6;157.6]	160.3 [157.0;164.6]	<0.001
Weight, kg	66.3 [57.0;76.9]	71.5 [65.0;79.7]	0.005	54.0 [46.2;59.3]	55.7 [50.6;62.8]	0.020
Body mass index, kg/m^2*^	23.8 [21.2;30.0]	23.3 [21.6;26.0]	0.111	23.2 [20.2;25.7]	21.4 [19.7;23.6]	0.006
Waist circumference, cm^*^	88.0 [78.0;97.5]	82.3 [77.2;89.4]	0.005	83.5 [77.0;92.0]	72.3 [67.4;79.3]	<0.001
Systolic blood pressure, mmHg	125.0 [120.5;135.0]	113.0 [106.0;122.0]	<0.001	120.0 [112.0;130.0]	104.0 [99.0;110.0]	<0.001
Diastolic blood pressure, mmHg	77.0 [73.0;85.0]	73.0 [68.0;81.0]	0.007	75.0 [67.0;82.0]	70.0 [64.0;75.0]	<0.001
Fasting plasma glucose, mg/dL	94.0 [89.0;99.0]	91.0 [87.0;96.0]	0.030	89.0 [86.0;96.0]	88.0 [84.0;93.0]	0.234
HbA1c, %	5.2 [ 5.1; 5.4]	5.3 [ 5.1; 5.5]	0.067	5.2 [ 5.0; 5.4]	5.3 [ 5.1; 5.5]	0.013
Total cholesterol, mg/dL	188.9 ± 34.2	180.7 ± 31.2	0.070	190.0 [167.0;217.0]	180.0 [158.0;202.0]	0.007
Triglyceride, mg/dL	114.0 [85.0;151.0]	104.0 [74.5;175.0]	0.503	108.0 [83.0;139.0]	71.0 [55.0;100.0]	<0.001
HDL cholesterol, mg/dL	55.0 [49.0;69.0]	47.3 [40.8;55.4]	<0.001	65.1 ± 16.1	58.7 ± 12.6	0.001
LDL cholesterol, mg/dL	109.0 [93.0;128.5]	109.0 [91.0;126.5]	0.832	113.0 [95.5;134.0]	103.0 [84.0;124.0]	0.058

Data are shown as mean ± standard deviation, median (interquartile range), or numbers (percentages). CAH, congenital adrenal hyperplasia; HDL, high-density lipoprotein; LDL, low-density lipoprotein; *Body mass index and waist circumference were not available in 12 and 54 patients with CAH.

The women with CAH (n = 93) showed a shorter stature, higher BMI, and higher waist circumference (P < 0.05) than the control women (n = 261). Blood pressure was also higher in the women with CAH than in the control women (P < 0.001). Fasting plasma glucose levels were similar between the two groups, but HbA1c was lower in women with CAH (P < 0.013). Total cholesterol, triglyceride, and HDL cholesterol levels were higher in the women with CAH than in the control women (P < 0.05).

We analyzed the metabolic outcomes of the CAH group versus the control group (**Table 2**). The CAH group had a two-fold higher risk of obesity than the control group among the women (OR [95% CI], 2.04 [1.18-3.50]), but the difference was not significant in the men. The increased risk of hypertension was found in CAH men (OR [95% CI], 2.67 [1.22-5.82]) but not in women. The risk of dyslipidemia and hyperglycemia did not differ between the two groups in either sex.

**Table 2:** Metabolic risks in CAH adults compared with age- and sex-matched controls.

	Men	Women
Obesity*	1.58 (0.89-2.80)	2.04 (1.18-3.50)
Dyslipidemia	0.81 (0.44-1.44)	1.17 (0.61-2.14)
Hyperglycemia	0.81 (0.42-1.51)	1.25 (0.70-2.17)
Hypertension	2.67 (1.22-5.82)	1.84 (0.78-4.17)

Data are shown as odds ratios (95% confidence interval). CAH, congenital adrenal hyperplasia; OR, odds ratio. *BMI was not available in 12 patients.

### Comparison According to Whether CAH Patients Have Metabolic Comorbidities

**Figure 1:**
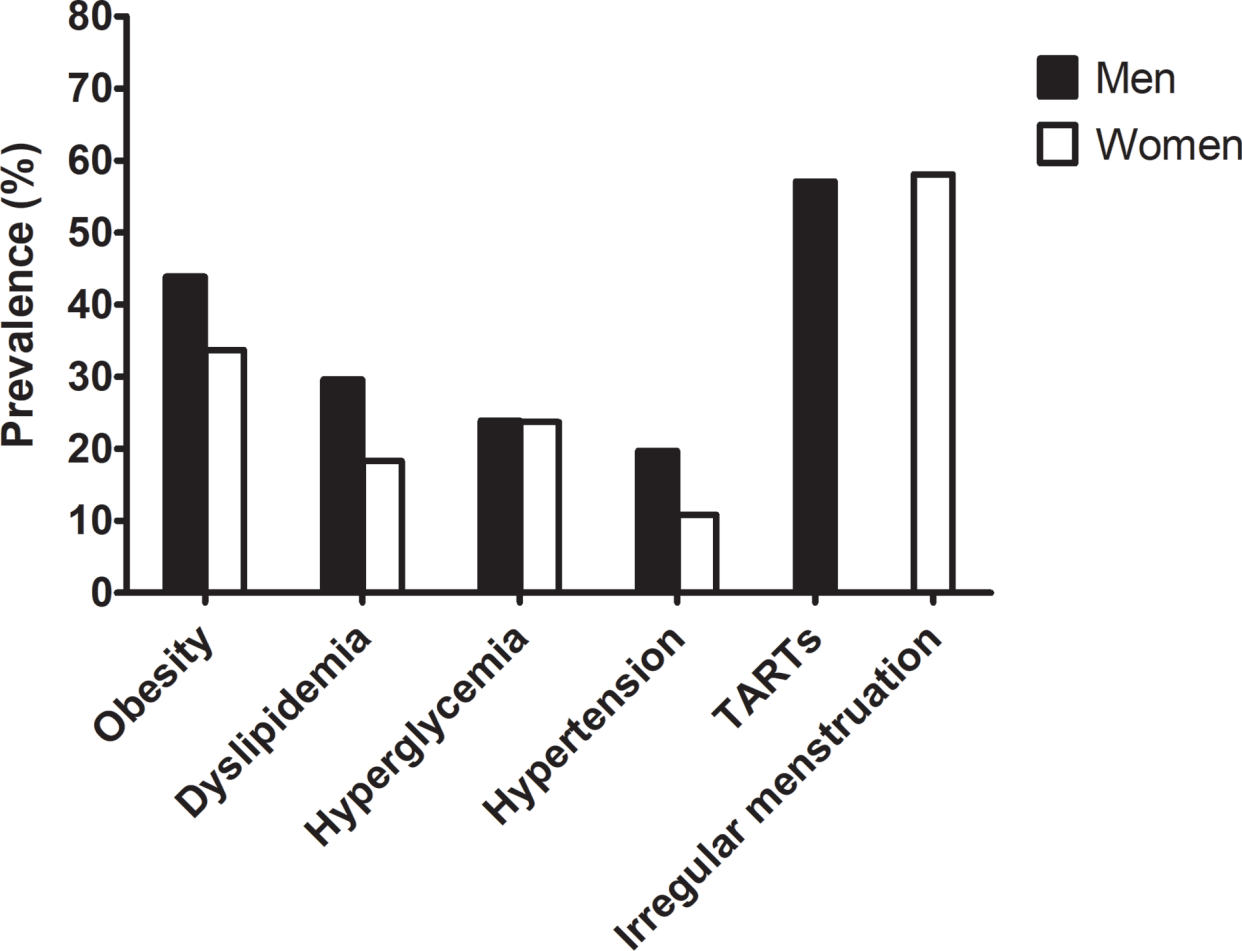
Prevalence of metabolic risk and gonadal dysfunction in the adult CAH patients according to sex.

**Figure 1** shows the prevalence of comorbidities in adults with CAH. The prevalence of any metabolic comorbidity was significantly higher in men than in women (70.4% *vs*. 52.7%, P = 0.032) without sex differences for each component. The prevalence of obesity, dyslipidemia, hyperglycemia, and hypertension was 43.9%, 29.6%, 23.9%, 19.7% in men and 33.7%, 18.3%, 23.7%, 10.8% in women, respectively. The number of patients with diabetes was only 6 in women and 2 in men with CAH. There was no history of gestational diabetes, cardiovascular or cerebrovascular disease.

We further analyzed the differences in hormone status and treatment regimen according to whether CAH patients were obese (**Table 3**). Neither age at diagnosis nor proportion of subtypes differed between obese and non-obese patients. Obese adults with CAH had a higher waist circumference and percentage of fat mass in both sexes but a lower percentage of lean mass than those without (P < 0.05 for all for both sexes). Adrenal limb thickness was significantly higher in obese men compared to non-obese men (P< 0.001). Although the difference of adrenal limb thickness was marginally significant between obese and non-obese women, adrenal tumors was more frequently found in obese women compared to non-obese women (P=0.019). Among the hormones, 17-OHP and DHEAS levels were significantly higher in obese women than in non-obese ones (P< 0.001, and P=0.009, respectively). All obese women had a 17-OHP level ≥ of 10 ng/mL. However, the glucocorticoid regimen and dose were not related to obesity in either sex. The number of patients with 0, 1, and ≥2 metabolic comorbidities was 29.6% (n=21), 35.2% (n=25), and 35.2% (n=25) in men with CAH, and 47.3% (n=44), 26.8% (n=25), and 25.8% (n=24) in women with CAH, respectively. No significant relationships of age at diagnosis, proportion of subtypes, and glucocorticoid doses with number of metabolic comorbidities were found in both sex (data not shown).

**Table 3:** Hormone status and treatment regimens in CAH men and women according to the presence of obesity.

Variables	Men			Women		
	Obesity (-)	Obesity (+)	*P*	Obesity (-)	Obesity (+)	*P*
	(N = 37)	(N = 29)		(N = 57)	(N = 29)	
Age at evaluation, years	26.0 [23.0;31.0]	28.0 [24.0;37.0]	0.164	28.0 [22.0;35.0]	32.0 [24.0;41.0]	0.177
Age at diagnosis, years	1.0 [0.0; 7.0]	1.0 [0.0; 8.0]	0.520	2.0 [0.0; 10.0]	5.0 [1.0; 17.0]	0.149
Salt-wasting form, n (%)	21 (56.8%)	18 (62.1%)	0.854	23 (40.4%)	10 (34.5%)	0.768
Height, cm	163.9 [157.6;168.3]	163.2 [158.9;167.8]	0.846	152.5 [147.2;157.0]	155.0 [148.8;158.6]	0.419
Weight, kg	57.4 [53.2;61.0]	79.8 [73.5;87.1]	<0.001	50.2 [43.1;54.6]	63.9 [58.4;70.6]	<0.001
Body mass index, kg/m^2^	21.7 [20.6;23.4]	30.4 [28.1;31.5]	<0.001	21.0 ± 2.6	28.5 ± 3.5	<0.001
Waist circumference (cm)	80.0 ± 5.6	98.8 ± 6.6	<0.001	79.0 [75.0;87.0]	90.0 [84.0;105.0]	<0.001
Lean mass (kg)	24.7 [23.6;26.5]	28.7 [26.7;31.3]	0.001	18.4 [16.7;21.0]	23.3 [20.8;29.4]	<0.001
Percentage lean mass (%)	42.6 [40.3;46.4]	36.4 [34.3;38.4]	<0.001	36.9 [34.2;39.9]	33.0 [29.8;57.3]	0.054
Fat mass (kg)	15.4 ± 4.6	29.1 ± 6.5	<0.001	19.5 [16.2;22.2]	25.0 [22.5;35.5]	<0.001
Percentage fat mass (%)	25.0 ± 6.1	36.5 ± 5.8	<0.001	36.0 ± 6.2	41.1 ± 6.9	0.006
17-OHP, ng/mL	60.0 [31.1;83.9]	81.6 [56.2;105.3]	0.055	38.3 [20.3;60.7]	71.8 [44.3;109.0]	<0.001
17-OHP <10 ng/mL (%)	2 (5.4%)	1 (3.5%)	1.000	9 (15.8%)	0 (0.0%)	0.026
Total testosterone, ng/mL	4.3 [ 3.5; 6.5]	4.7 [ 3.7; 5.4]	0.838	0.5 [ 0.1; 0.9]	0.7 [ 0.5; 1.8]	0.138
DHEAS, ng/mL	736.0 [277.0;1351.0]	686.5 [295.5;1435.0]	0.639	292.0 [105.5;743.5]	632.0 [306.0;1690.0]	0.009
Plasma renin activity, ng/mL/hr	7.4 [ 3.4;10.4]	10.7 [ 5.4;21.4]	0.077	8.4 [ 4.2;11.9]	8.2 [ 4.8;15.4]	0.428
Plasma renin activity <3 ng/mL/hr	7 (20.0%)	7 (7.1%)	0.277	8 (14.3%)	2 (8.3%)	0.715
ACTH, pg/mL	117.2 [40.5;281.3]	232.6 [81.9;681.0]	0.164	33.1 [14.9;232.7]	70.2 [37.4;188.0]	0.228
Adrenal thickness on CT, mm	5.5 [ 4.1; 6.3]	7.4 [ 6.1; 8.9]	<0.001	5.8 [ 4.5; 7.3]	6.7 [ 5.3; 9.2]	0.064
Adrenal tumors, n (%)	3 (12.0%)	5 (26.3%)	0.262	1 (2.9%)	4 (28.6%)	0.019
Glucocorticoid regimen, n (%)			1.000			1.000
Hydrocortisone	1 ( 2.7%)	0 ( 0.0%)		5 ( 8.8%)	3 (10.3%)	
Prednisolone	36 (97.3%)	29 (100.0%)	0.850	52 (91.2%)	26 (89.7%)	0.400
Glucocorticoid dose, mg/day	30.0 [20.0;30.0]	30.0 [20.0;30.0]	0.850	30.0 [20.0;30.0]	25.0 [20.0;30.0]	0.400
Fludrocortisone use, n (%)	31 (83.8%)	22 (75.9%)	0.623	43 (75.4%)	14 (48.3%)	0.023

Data are shown as mean ± standard deviation, median [interquantile range], or numbers (percentages). BMI and adrenal thickness were not available in 12 and 75 patients. Body composition data were not available in 20 men and 38 women. 17-OHP, 17-hydroxyprogesterone; DHEAS, dehydroepiandrosterone sulfate.

### Comparison of Whether CAH Patients Had TARTs or Menstrual Irregularities

The prevalence of TARTs in men was 58.1% (**Figure 1** and **Table 4**). The presence of TARTs was not related to disease control, hormone status, glucocorticoid regimen, or adrenal limb thickness in men. Among the women, 57.1% exhibited menstrual irregularities (**Figure 1** and **Table 4**). Women with irregular periods showed a shorter stature and a higher DHEAS level than those with regular periods (P< 0.05 for both). Other hormones, steroid regimens, and adrenal limb thickness did not differ according to menstrual regularity.

**Table 4:** Hormone status and treatment regimen in men with CAH according to the presence of testicular adrenal rest tumors (n = 70) and women with CAH according to menstruation (n = 86).

Variables	Men			Women		
	TART (-)	TART (+)	*P*	Irregular	Regular	*P*
	(N = 30)	(N = 40)		(n = 50)	(n = 36)	
Age at evaluation, years	27.0 [22.0;35.0]	27.0 [24.0;31.0]	0.957	29.5 [22.0;39.0]	27.0 [22.5;34.5]	0.277
Height, cm	164.6 ± 8.2	162.2 ± 6.1	0.196	151.0 [146.4;156.5]	155.3 [151.6;159.7]	0.011
Weight, kg	66.0 [56.8;76.4]	67.4 [57.4;79.8]	0.822	54.4 [45.6;58.6]	52.0 [44.7;59.8]	0.601
Body mass index, kg/m^2^	24.0 [21.4;28.1]	23.8 [21.7;30.6]	0.474	23.2 [20.2;25.6]	22.5 [20.1;25.8]	0.768
Waist circumference (cm)	89.9 ± 11.1	88.5 ± 11.5	0.669	87.0 [78.0;93.0]	80.5 [75.0;90.0]	0.136
Lean mass (kg)	26.9 [24.0;30.5]	26.2 [24.5;29.1]	0.702	20.7 [17.9;23.1]	19.5 [17.4;21.9]	0.788
Percentage lean mass (%)	39.8 [37.6;45.5]	39.6 [34.8;42.8]	0.371	35.1 [32.2;38.3]	36.5 [33.0;41.6]	0.345
Fat mass (kg)	22.3 ± 7.9	21.9 ± 9.7	0.865	21.1 [18.8;25.5]	21.6 [16.2;24.9]	0.570
Percentage fat mass (%)	31.3 ± 7.4	30.2 ± 8.9	0.657	39.0 ± 6.9	37.0 ± 5.9	0.270
17-OHP, ng/mL	58.2 [20.7;85.9]	72.5 [48.6;100.9]	0.098	49.5 [28.5;97.4]	45.3 [27.1;68.0]	0.203
17-OHP <10 ng/mL	3 (10.0%)	1 (2.5%)	0.307	4 (8.0%)	5 (13.9%)	0.482
Total testosterone, ng/mL	4.2 [ 3.3; 5.4]	4.8 [ 3.7; 6.2]	0.314	0.7 [ 0.3; 1.6]	0.5 [ 0.1; 0.7]	0.086
DHEAS, ng/mL	885.5 [285.0;1396.0]	584.0 [302.0;1348.0]	0.963	462.5 [199.0;1559.0]	251.0 [102.5;573.5]	0.013
Plasma renin activity, ng/mL/hr	8.1 [ 4.2;12.3]	7.3 [ 3.8;19.1]	0.748	8.6 [ 4.6;14.2]	7.4 [ 4.7;11.2]	0.458
Plasma renin activity <3 ng/mL/hr	3 (10.0%)	7 (18.9%)	0.493	5 (10.9%)	4 (12.5%)	1.000
ACTH, pg/mL	82.5 [42.4;699.2]	187.0 [46.9;318.0]	0.948	41.0 [16.0;125.8]	58.9 [35.5;241.6]	0.188
Adrenal thickness on CT, mm*	6.2 [ 5.4; 7.4]	6.1 [ 4.1; 7.2]	0.512	6.7 [ 5.2; 8.4]	6.0 [ 4.5; 6.9]	0.202
Adrenal tumors, n (%)	4 (20.0%)	4 (16.7%)	1.000	4 (14.8%)	1 (5.3%)	0.387
Glucocorticoid regimen, n (%)			1.000			0.649
Hydrocortisone	1 (3.3%)	2 (5.0%)		3 (6.0%)	4 (11.1%)	
Prednisolone	29 (96.7%)	38 (95.0%)		47 (94.0%)	32 (88.9%)	
Glucocorticoid dose, mg/day	30.0 [20.0;30.0]	30.0 [20.0;30.0]	0.736	30.0 [20.0;30.0]	27.5 [20.0;30.0]	0.379
Fludrocortisone use, n (%)	24 (80.0%)	32 (80.0%)	1.000	29 (58.0%)	27 (75.0%)	0.161

Data are shown as mean ± standard deviation, median (interquartile range), or numbers (percentages). 17-OHP, 17-hydroxyprogesterone; DHEAS, dehydroepiandrosterone sulfate. ^*^Adrenal thickness was available in 43 men and 50 women.

## Discussion

The present study suggested that waist circumference and blood pressure were higher in adults with CAH compared with age- and BMI-matched controls from the KNHANES database. Moreover, there was an increased risk of hypertension in men and obesity in women with CAH than controls. Obese men with CAH showed higher adrenal limb thicknesses than non-obese ones, while obese women had higher 17-OHP and DHEAS levels than non-obese ones. In addition, more than half of the adults with CAH exhibited TARTs or menstrual irregularities. The presence of TARTs in men with CAH was not related to disease control, while women with CAH who had irregular periods were shorter and had higher DHEAS levels than those with regular periods.

The prevalence of obesity or central obesity in adults with classic CAH is reportedly 20–50%, higher than that in healthy controls (3–6). In the present study, obesity was more prevalent in women with CAH, and waist circumference was obviously higher in both sexes than in healthy controls, consistent with the increased abdominal obesity and higher visceral to subcutaneous fat ratio linked to insulin resistance and inflammation, suggesting an unhealthy metabolic phenotype among adults with CAH (18).

It remains controversial whether hypertension, dyslipidemia, and hyperglycemia are more prevalent in adults with CAH than in healthy controls. The prevalence of hypertension was higher in the NIH and Swedish nationwide CAH cohorts (4, 5, 8) and similar or lower in the CaHASE and French cohorts (3, 19). In our study, men with CAH but not women with CAH showed a higher prevalence of hypertension than the controls, which can be related to the relatively high proportion of fludrocortisone use and central obesity among men with CAH and the protective effect of estrogen on blood pressure among women with CAH. The prevalence of dyslipidemia and hyperglycemia was similar between adults with CAH and controls in this study. However, fasting glucose and HDL cholesterol levels in men with CAH and total cholesterol, triglyceride, and HDL cholesterol levels in women with CAH were higher than the values in controls, although a recent meta-analysis including 300 pediatric and 137 adults showed no difference in glucose and lipid levels (8). The result of higher HDL-cholesterol levels in our patients with CAH remains to be reproduced in further studies, and the underlying mechanism also remains to be determined. However, Falhammar et al. suggested that older women with CAH had also higher lean mass which may explain the favorable lipid profile (20). Our study population mainly consisted of young adult patients who showed elevated androgen levels, suggesting poor disease control. Inconsistency in reporting the prevalence of metabolic comorbidities may have resulted from differences in patient age, glucocorticoid and mineralocorticoid doses, treatment target goals, disease control status, and 21-hydroxylase activity.

We further analyzed factors that differed according to whether patients with CAH had more metabolic risk factors. Previous studies identified that adults with CAH are at risk for hyperandrogenism and iatrogenic hypercortisolism, both of which lead to obesity, insulin resistance, and cardiometabolic comorbidities (5, 6, 9, 10). In addition to glucocorticoid overtreatment, suppressed 17-OHP levels were related to obesity or metabolic morbidity (3, 11) as well as androgen excess, suggesting that poor disease control contributed to higher metabolic risk factors as shown in our study.

Intriguingly, obese men with CAH presented with thick adrenal limbs, while obese women had high 17-OHP and DHEAS levels. Few studies have examined the adrenal morphology of adults with CAH (6, 21). Adrenal nodules were more frequently detected in poorly controlled patients with CAH, and regressed in size after high-dose steroids (13). Adrenal hyperplasia and/or nodules have been reported in 29.3– 45% of patients with CAH (22), with significant correlation with high levels of ACTH, 17-OHP, and plasma renin activity (6). Adrenal tumors were detected in 15% (14/93) among our adult patients evaluated by CT. Despite the limitation of a single hormonal measurement in the morning due to the cross-sectional nature of this study, adrenal limb thickness or the presence of adrenal tumors could be a long-term disease control marker in adults with CAH.

Obese women with CAH showed higher 17-OHP levels than non-obese ones, suggesting the contribution of androgen excess. A similar phenomenon was demonstrated in polycystic ovary syndrome in postmenopausal women (23–25). Androgen excess drives visceral fat accumulation and induces the android phenotype in women with CAH (26, 27). Even with continued treatment, patients may respond poorly to glucocorticoid treatment (6, 28) related to glucocorticoid receptor gene polymorphism in addition to low adherence to glucocorticoid therapy regimens (29). Further studies are warranted to determine optimal targets and androgen metabolites that reflect adequate hormonal control and ideal glucocorticoid regimens to minimize cardiometabolic comorbidities.

The presence of TART is the main factor of male infertility since it blocks the seminiferous tubules and leads to Leydig cell failure, which has a reported prevalence of around 37% (14–86%) depending on age and genotype (5, 6, 19, 30). In our study, TARTs were found in 57.1% of men with CAH who underwent testicular sonography. Since TARTs express adrenal-specific enzymes and ACTH and angiotensin II receptors (31), poor disease control and high ACTH or renin activity can lead to adrenocortical hyperplasia and TARTs (5, 32, 33), although the lack of a correlation between 17-OHP levels and TARTs has been reported (6, 12, 34). Although the present study failed to demonstrate a relationship between disease control and the presence of TARTs, in our previous study, TART size was related to the undertreatment percentage (35).

The prevalence of menstrual irregularity was up to 58.1% in the women with CAH in our study, which is consistent with that of a previous report of 30–75% depending on genotype (3). Women with CAH and irregular periods exhibited shorter stature and higher DHEAS levels, reflecting hyperandrogenism. Patients with CAH exposed to high androgens in childhood can experience earlier epiphyseal closure and short stature. Hyperandrogenism suppresses serum progesterone, which reduces luteinizing hormone pulsatility during the follicular phase and induces endometrial thinning, leading to oligo- or anovulation and oligo- or amenorrhea (36–38).

This cross-sectional study had several limitations. There were several missing values due to the retrospective study design. We could not assess the total cumulative doses of glucocorticoid or perform a longitudinal hormone assessment to determine disease control due to a lack of childhood data before the electronic medical record era. Since most patients were diagnosed before the genetic testing era, we could not analyze the patients’ clinical or hormone characteristics according to genotype. When we assessed single hormone measurements in the morning at the last visit, only 7.3% of patients had well-controlled disease based on the early-morning 17-OHP levels. Since undertreatment still affected most of our patients compared to those in previous reports (3), we could not compare the effect of undertreatment versus overtreatment on long-term outcomes. Androstenedione, which was known to be the reliable marker for disease control (1), was not checked due to the cost and unavailability. Although this cross-sectional design could not prove the causal relationship of disease itself or treatment effect with metabolic complications, our study had several strengths. First, it is the largest single-center Asian study of adults with CAH. We also compared age-, sex-, and ethnicity-matched controls to assess metabolic risk. We separately analyzed sex-specific indicators according to health outcomes and obtained different findings by sex. Adrenal thickness assessed by CT was incorporated into the variables to reflect long-term glucocorticoid exposure. Most patients had taken the same dosage of prednisolone in adulthood, thereby allowing us to compare their hormonal effects in patients with CAH without glucocorticoid dose interference.

## Conclusion

The present study confirmed the higher prevalence of metabolic morbidities in young adults with CAH than in healthy controls. Moreover, adverse metabolic outcomes and menstrual irregularity were associated with poor disease control in our study subjects. Considering the harmful effects of poor disease control on long-term outcomes, compliance should be guaranteed. Early attention should be paid to identifying at-risk patients and regular follow-up provided to minimize cardiovascular risk in patients with CAH. Further studies are needed to elucidate the mechanisms leading to metabolic morbidity and the respective roles of androgen excess and glucocorticoid exposure.

## Data Availability Statement

The original contributions presented in the study are included in the article/supplementary files. Further inquiries can be directed to the corresponding authors.

## Ethics Statement

The studies involving human participants were reviewed and approved by Seoul National University Hospital Institutional Review Board. Written informed consent for participation was not required for this study in accordance with the national legislation and the institutional requirements.

## Author Contributions

SL and JK contributed in the conception of the work, participated in the study design, and wrote the manuscript. YL contributed in the conception of the work, participated in the study design, and critically revised the manuscript. HJ, SK, CA, SK, and CS contributed in the conception of the work, performed statistical analyses, and critically revised the manuscript. All authors contributed to the article and approved the submitted version.

## Funding

This study was funded by a donation from one CAH patient.

## Conflict of Interest

The authors declare that the research was conducted in the absence of any commercial or financial relationships that could be construed as a potential conflict of interest.

## Publisher’s Note

All claims expressed in this article are solely those of the authors and do not necessarily represent those of their affiliated organizations, or those of the publisher, the editors and the reviewers. Any product that may be evaluated in this article, or claim that may be made by its manufacturer, is not guaranteed or endorsed by the publisher.
